# Brain Functional Alterations in Prepubertal Boys With Autism Spectrum Disorders

**DOI:** 10.3389/fnhum.2022.891965

**Published:** 2022-05-19

**Authors:** Xipeng Yue, Ge Zhang, Xiaochen Li, Yu Shen, Wei Wei, Yan Bai, Yu Luo, Huanhuan Wei, Ziqiang Li, Xianchang Zhang, Meiyun Wang

**Affiliations:** ^1^Department of Medical Imaging, Zhengzhou University People’s Hospital & Henan Provincial People’s Hospital, Zhengzhou, China; ^2^Academy of Medical Sciences, Zhengzhou University, Zhengzhou, China; ^3^Henan Provincial People’s Hospital, Xinxiang Medical University, Xinxiang, China; ^4^MR Collaboration, Siemens Healthineers Ltd., Beijing, China

**Keywords:** autism spectrum disorders, amplitude of low-frequency fluctuation, regional homogeneity, resting-state fMRI, neural activity

## Abstract

**Objectives:**

Abnormal brain function in ASD patients changes dynamically across developmental stages. However, no one has studied the brain function of prepubertal children with ASD. Prepuberty is an important stage for children’s socialization. This study aimed to investigate alterations in local spontaneous brain activity in prepubertal boys with ASD.

**Materials and Methods:**

Measures of the amplitude of low-frequency fluctuations (ALFF) and regional homogeneity (ReHo) acquired from resting-state functional magnetic resonance imaging (RS-fMRI) database, including 34 boys with ASD and 49 typically developing (TD) boys aged 7 to 10 years, were used to detect regional brain activity. Pearson correlation analyses were conducted on the relationship between abnormal ALFF and ReHo values and Autism Diagnostic Observation Schedule (ADOS) and Autism Diagnostic Interview-Revised (ADI-R) scores.

**Results:**

In the ASD group, we found decreased ALFF in the left inferior parietal lobule (IPL) and decreased ReHo in the left lingual gyrus (LG), left superior temporal gyrus (STG), left middle occipital gyrus (MOG), and right cuneus (*p* < 0.05, FDR correction). There were negative correlations between ReHo values in the left LG and left STG and the ADOS social affect score and a negative correlation between ReHo values in the left STG and the calibrated severity total ADOS score.

**Conclusion:**

Brain regions with functional abnormalities, including the left IPL, left LG, left STG, left MOG, and right cuneus may be crucial in the neuropathology of prepubertal boys with ASD. Furthermore, ReHo abnormalities in the left LG and left STG were correlated with sociality. These results will supplement the study of neural mechanisms in ASD at different developmental stages, and be helpful in exploring the neural mechanisms of prepubertal boys with ASD.

## Introduction

Autism spectrum disorder (ASD) is a neurodevelopmental condition characterized by abnormal social interactions and development, repetitive behaviors, and limited interests (Philip et al., 2012). The latest large-scale global survey estimated that the incidence rate of ASD is approximately 1-2% and that it has been steadily increasing (Lai et al., 2015). However, the pathogenetic mechanisms of ASD remain unknown.

In recent years, the development of neuroimaging has provided a new platform for the study of neural changes in ASD, and emerging evidence reveals that neural function in patients with ASD changes dynamically with age (Van Rooij et al., 2018; Haghighat et al., 2021). For example, by limiting the subjects to infants (2 to 3 years old), research by Redcay (Redcay and Courchesne, 2008) showed that ASD participants had greater activation primarily within the right and medial frontal regions based on fMRI. In preschool boys with ASD between 3 and 6 years old, Lan (Lan et al., 2021) found increased ReHo in the right calcarine as well as decreased ReHo in the opercular part of the left inferior frontal gyrus, the left middle temporal gyrus, the left angular gyrus, and the right medial orbital frontal cortex. In a study by Shukla et al. (2010), ReHo was lower in the adolescent ASD between the ages of 10 and 18 than in the TD group in the superior parietal and anterior prefrontal regions. Very obviously, the imaging results of brain function in ASD patients at different developmental stages are not consistent, and limiting the age of subjects to a specific period of development is a good way to reduce the influence of a larger age span on brain function changes in patients with ASD (Nomi and Uddin, 2015). Nevertheless, no studies have been conducted on brain function imaging of ASD in the prepubertal period.

Prepuberty is a time window between the preschool period and the adolescent period and is a preparatory stage for the development of adolescence (Stormshak et al., 2011). Moreover, it represents an important stage for children to improve their sociality in terms of their adaptability, adjustment, and reactivity. A study showed that orienting attention seems to improve from 6 or 7 years onward by gaining the ability to disengage attention when necessary (Wainwright and Bryson, 2005). The ability to cope with these multi-dimensional switching tasks improves greatly between 7 and 9 years of age (Anderson et al., 2000). Neurological development in 7- to 10-year-old children is essential for responses to social evaluation (Achterberg et al., 2017). All those factors allow better integration into society. Therefore, it is necessary to explore the abnormal neurological changes in prepuberty.

In this study, we conducted amplitude of low-frequency fluctuations (ALFF) and regional homogeneity (ReHo) analysis based on resting-state functional magnetic resonance imaging (RS-fMRI) data from the Autism Brain Imaging Database Exchange (ABIDE) in children (boys;7–10 years old) to specifically explore alterations in local spontaneous activity in prepubertal children with ASD and analyze correlations with clinical manifestations. This study will supplement the study of brain function in ASD at different periods of development, which will be helpful to understand neural mechanisms from the perspective of functional brain imaging.

## Materials and Methods

### Participants

We selected 34 male children with ASD and 49 male typically developing (TD) control participants matched for age from six research centers in the ABIDE database (site: Yale, UCLA, KKI, UM, NYU, STANFORD). The data was obtained from the public database. According to the policy of the institutional ethical review board, this kind of research is waived for ethical review. Structural and functional MRI images of every participant were acquired from ABIDE. The acquisition parameters, informed consent, diagnostic criteria and specific protocols used at every site are available on the database website^1^. The data conditions of the ASD group and the TD group in the database were as follows:(1) males with DSM-IV-TR diagnosis;(2) ages ranging from 7-10 years of age; (3) subjects with complete behavioral data provided by the database, including ADOS (Social Affect Score, Restricted and Repetitive Behavior Score, Calibrated Severity Total Score) and ADI-R (Reciprocal Social Interaction Subscore, Abnormalities in Communication Subscore, Restricted, Repetitive, and Stereotyped Patterns of Behavior Subscore) and (4) all the subjects were right-handed.

To evaluate the impact of multi-site data, we numbered the sites as 1,2,3,4,5,6 in this order: Yale, UCLA, KKI, UM, NYU and STANFORD. The subject in each imaging site is assigned the corresponding number (site code). Then the Rank sum test (Mann-Whitney U test) was used to compare site codes between ASD group and HC group. The result showed that there was no significant difference between the two groups (*Z* = −0.251, *p* = 0.802). So, we thought that multi-center data has no influence on the results of this study.

### Functional MRI and ALFF and ReHo Analyses

Resting-state fMRI data were processed using the Resting-State fMRI Data Analysis Toolkit plus V1.25 (RESTplus V1.25) (Yan et al., 2016). The preprocessing included the following steps: (1) removing the first 10 time points to eliminate the interference from magnetization and participants. (2) slice timing correction with the middle slice as a reference; (3) head motion correction and realignment (subjects presenting maximal translation in 3 directions > 3 mm or maximal rotation > 3° were excluded from further analyses); (4) spatial normalization to the Montreal Neurological Institute (MNI) space (voxel size = 2 × 2 × 2 mm); (5) spatial smoothing with a 6 × 6 × 6 mm full-width at half maximum (FWHM) Gaussian kernel (just used before ALFF calculation); (6) linear detrending was removed to reduce the influence of MRI equipment; (7) regressing out the nuisance covariates including mean signals from white matter and cerebrospinal fluid as well as the head motion effect with the Friston 24-parameter model; (8) temporal bandpass filtering (0.01–0.08 Hz, just used before the ReHo calculation).

RESTplus V1.25 was utilized for ALFF and ReHo calculations (Yan et al., 2016). After data preprocessing, the ALFF value was calculated in accordance with the following steps: the fast fourier transform algorithm was used to transform the data to the frequency domain; the power spectrum was obtained and the square root in the spectrum and the average of the square root between 0.01-0.08 Hz in each voxel were calculated as ALFF values. The original ALFF values were transformed to z scores for group comparison.

The ReHo value was calculated by reducing low-frequency drift and high-frequency noise, both of which were performed by passing the spatially standardized data through a 0.01–0.08 Hz bandpass filter. Similarity between a single voxel and the surrounding 27 voxels was calculated based on ReHo using Kendall’s coefficient of concordance (KCC). The ReHo value of each voxel was transformed to a z score for standardization purposes. Finally, a smoothing core of 6 mm was used for spatial smoothing after ReHo calculation.

### Statistical Analysis

Two independent-sample, non-parametric tests (Mann-Whitney U test) were used to assess the age and full-scale IQ difference between boys with ASD and TDs. To explore the differences in ReHo and ALFF between boys with ASD and TDs, the RESTplus statistical analysis toolkit was used to conduct a two-sample t-test. *p* < 0.05 was considered statistically significant with false discovery rate (FDR) correction. To determine the relationship between the brain regions with significantly altered ALFF or ReHo values and the ADOS, ADI-R, Pearson correlation analysis was performed in SPSS 20.0 software (*p* < 0.05). To identify the association between ALFF and ReHo in abnormal brain functional regions, the ALFF and ReHo values of brain regions with abnormal brain functional alterations were individually extracted, and Pearson correlation analysis was conducted.

## Results

### Demographic and Clinical Characteristics

All of the data for the ASD and TD groups were retained after processing. There was no significant difference in age or full-scale IQ between the two groups. The ADOS and ADI-R values for the ASD group were consistent with the ASD standard, as shown in Table 1. TD subjects were not measured by the corresponding scale.

**TABLE 1:** Demographic and clinical characteristics of boys with ASD and TD participant.

	ASD group (*n* = 34)	TD group (*n* = 49)	Z	*P*
Age	9.25 ± 1.17	9.36 ± 1.14	−0.713	0.476
full-scale IQ	101.33 ± 17.03	107.62 ± 11.75	−1.908	0.056
ADOS				
Social Affect Score	8.59 ± 4.61			
Restricted and Repetitive Behavior Score	3.15 ± 2.19			
Calibrated Severity Total Score	6.50 ± 3.22			
ADI-R				
Reciprocal Social Interaction Subscore	20.82 ± 6.45			
Abnormalities in Communication Subscore	16.00 ± 5.30			
Restricted, Repetitive, and Stereotyped Patterns of Behavior Subscore	6.24 ± 2.64			

*Data are mean ± standard deviation. ADOS, Autism Diagnostic Observation Schedule; ADI-R, Autism Diagnostic Interview—Revised.*

### Differences in ALFF/ReHo Between the ASD and TD Groups

**FIGURE 1:**
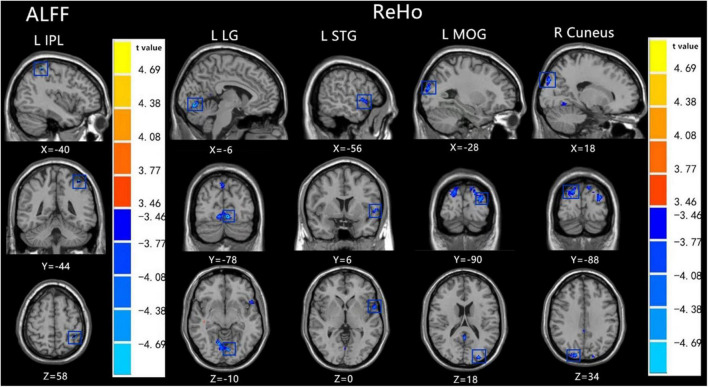
Brain regions with abnormal ALFF **(left)** and ReHo **(right)** between the ASD group and the TD group based on t tests. Blue colors denote decreased ALFF value in L IPL and decreased ReHo values in L LG, L STG, L MOG, and R Cuneus. The blue transparent box shows the corresponding brain region. The color bars indicate the t value (FDR correction, *p* < 0.05). L IPL: left inferior parietal lobule, L LG: left lingual gyrus, L STG: left superior temporal gyrus, L MOG:left middle occipital gyrus. X,Y,Z is coordinates of primary peak locations of the abnormal brain region in the Montreal Neurological Institute(MNI) space.

**FIGURE 2:**
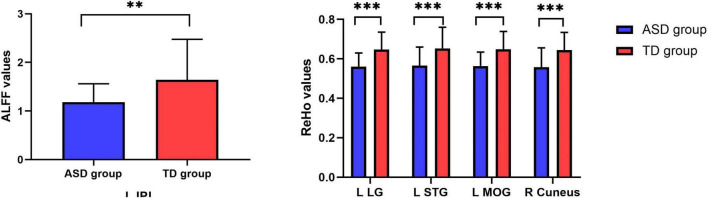
ALFF/ReHo signal values for altered regional brain regions between boys with ASD and TDs. _**_means *p* < 0.01, _***_ means *p* < 0.001.

RS-fMRI analysis showed that, compared with TD participants, boys with ASD had decreased ALFF values in the left inferior parietal lobule (IPL) as well as decreased ReHo values in the left lingual gyrus (LG), left superior temporal gyrus (STG), left middle occipital gyrus (MOG), and right cuneus (FDR correction, *p* < 0.05) (Tables 2, 3 and Figures 1, 2).

**TABLE 2:** ALFF differences between the ASD group and TD group.

Brain region	Cluster size	MNI coordinates			AAL	Peak *T*-value
		X	Y	Z		
L IPL	27	−40	−44	58	Parietal_Inf_L	−5.37

*L, left; R, right; IPL, inferior parietal lobule; MNI, Montreal Neurological Institute; ALFF, amplitude of low-frequency fluctuations; AAL, anatomical automatic labeling.*

**TABLE 3:** ReHo differences between the ASD group and TD group.

Brain region	Cluster size	MNI coordinates			AAL	Peak T-value
		X	Y	Z		
L LG	103	−6	−78	−10	Lingual_L	−5.22
L STG	52	−56	6	0	Temporal_Pole_Sup_L	−4.3545
L MOG	79	−28	−90	18	Occipital_Mid_L	−4.6258
R Cuneus	52	18	−88	34	Occipital_Sup_R	−4.4592

*L, left; R, right; LG, lingual gyrus; STG, superior temporal gyrus; MOG, middle occipital gyrus; MNI, Montreal Neurological Institute; ReHo, regional homogeneity; AAL, anatomical automatic labeling.*

### Correlations Between ALFF/ReHo Values in Abnormal Regions With ADOS and ADI-R

**FIGURE 3:**
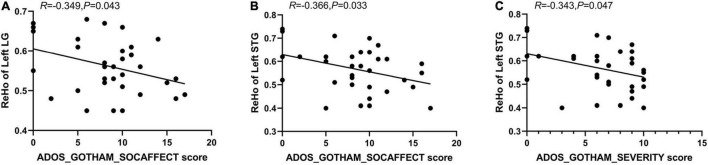
Negative correlation between ReHo values of left LG with ADOS_GOTHAM_SOCAFFECT score **(A)**, and between ReHo values of left STG with ADOS_GOTHAM_SOCAFFECT score **(B)**; Negative correlation between the ReHo values of left STG with ADOS_GOTHAM_SEVERITY score **(C)**.

The results of Pearson correlation analyses revealed a negative correlation between ReHo values in the left LG and left STG with the social affect score of the ADOS (i.e., ADOS_GOTHAM_SOCAFFECT score) (*R* = -0.349, *p* = 0.043; *R* = -0.366, *p* = 0.033); there was a negative correlation between ReHo values in the left STG and the calibrated severity total score of the ADOS (i.e., ADOS_GOTHAM_SEVERITY score) (*R* = -0.343, *p* = 0.047) (Figure 3). However, we did not find a significant correlation between ALFF values in IPL with ADOS and no significant correlation between ALFF/ReHo values in any of the five abnormal brain regions and ADI-R was found.

### Correlations Between ALFF and ReHo Values in Abnormal Regions

The results of Pearson correlation analyses revealed that no correlations were detected in the left IPL, left LG, left STG, left MOG, and right cuneus between the ALFF and ReHo values (*R* = -0.020, *p* = 0.909; *R* = -0.261, *p* = 0.137; *R* = 0.242, *p* = 0.167; *R* = 0.067, *p* = 0.705; *R* = 0.071, *p* = 0.81).

## Discussion

In this study, based on RS-fMRI data from a multicenter sample, we used ALFF/ReHo analyses to explore alterations in spontaneous neural activity in the brains of prepubertal boys with ASD. The results show that ALFF values in the left IPL were decreased in the ASD group. ReHo values in the left LG, left STG, left MOG, and right cuneus were also decreased in the ASD group. Correlation analyses revealed that these abnormal ReHo values in the left LG and left STG were associated with the sociality reflected by the ADOS score.

We found that ALFF values of the left IPL decreased, suggesting that there was a decrease in the intensity of regional brain activity. The left IPL has been consistently shown to have a key role in the regulation of motor skills (Bohlhalter et al., 2009). Evidence from both humans (Wymbs and Grafton, 2015) and non-human primates (Quallo et al., 2009) suggest that the left IPL is also involved in the formation of skill-specific representations during skill learning. While primarily associated with motor skills, the left IPL also plays a major role in social cognition (Igelström and Graziano, 2017), as it may support the development of social skills, including the theory of mind and empathy (Deramus and Kana, 2015). In fact, abnormal IPL activity in ASD patients at different developmental stages has been frequently reported in previous studies. Kana (Kana et al., 2017) found that adolescent ASD patients had significantly reduced activity in the left IPL by using RS-fMRI. Mitelman et al. (2018) revealed that adult subjects with ASD showed decreased metabolic rates in the IPL. However, in the study of Redcay and Courchesne (2008), the authors did not find that infants with ASD (2–3- year-old children) had reduced activity in the left IPL; there was also no reduced activity in left IPL in preschool boys with ASD (Ventola et al., 2015). In the present study, the results showed decreased brain activity in the left IPL in prepubertal boys with ASD. Therefore, we hypothesized that the decreased brain activity of the left IPL in ASD patients may start from pre-puberty and then participate in the process influencing the sociality of ASD patients.

Simultaneously, we found that there was a decrease in synchronization of neural activity reflected by ReHo in the left LG in pre-pubertal boys with ASD. The LG has been suggested to be involved in visual recognition and episodic memory consolidation (Kukolja et al., 2016) and has been considered to play a role in the process of perceiving the surrounding environment (Bischof and Bassetti, 2004). However, in a study of preschool (Lan et al., 2021) and adolescent children (Shukla et al., 2010) with ASD, decreased ReHo in the left LG was not found. Furthermore, the decrease in synchronization of neural activity in the left LG that we found had a negative correlation with the ADOS social affect score. Therefore, we speculate that decreased ReHo in the left LG may be a unique brain function change in prepubertal children with ASD, and these decreases are closely related to the abnormal social emotion development observed in pre-pubertal ASD patients.

In this study, decreased local synchronization of neuronal activity in the left STG was also found in patients with ASD. This is similar to Wu’s findings (Wu et al., 2020). The STG is considered a key structure involved in cognitive processing (Riecke et al., 2017; Marrero et al., 2020). It plays an important role in emotional processing (Peelen et al., 2010), decision-making (Yang et al., 2016), and reward processing (Vrieze et al., 2013). Some researchers have found that decreased local synchronization reflected by ReHo in the STG could be attributed to impaired cognitive function (Zhang et al., 2012). The STG is one of the hot brain regions studied in ASD. The study of Wu revealed morphological alterations of the STG among preschool and school-aged children with ASD (Wu et al., 2020). Abnormalities in the STG were also found in adults with ASD by fMRI (Wang et al., 2019). Our study complements previous studies and indicates that abnormal STG is present at different stages of development in individuals with ASD. Additionally, ReHo values in the left STG had a negative correlation with the social affect score and severity scores of the ADOS. Social affective disorder is the most severe symptom observed in autism patients (Thom et al., 2021), with the highest proportion observed across ADOS scores (Bal et al., 2020). Thus, we speculate that the decreased local synchronization of neural activity in the left STG may be related to the social emotions of ASD children in prepuberty, further affecting the overall symptom score.

Decreased local synchronization of neuronal activity in the left MOG was observed in this study. This is similar to the results of Guo’s research, which showed that for ASD patients in prepuberty and adolescence, the decrease in ALFF was significant in the left MOG (Guo et al., 2017). On the one hand, as the core area of the visual network (VN), the MOG is mainly responsible for regulating visual function, which is essential for social cognitive function (Tohid et al., 2015). Researchers have shown that deficits in MOG potentially could be associated with body image distortion (Su et al., 2021). Another study revealed that patients with mild cognitive impairment also showed hypoperfusion in the occipital gyrus (Arslan et al., 2020). On the other hand, abnormalities in sensory perception, including visual function have been invoked as possible primary causes of some characteristic features of ASD (Dakin and Frith, 2005). These results may indicate that abnormalities in the Left MOG play an important role in different developmental stages of ASD.

We also observed that the ASD patients had significantly decreased ReHo in the right cuneus. The cuneus receives visual information from the retina of the same-sided superior quadrant and is involved in basic visual processing (Liao et al., 2019). Many studies have reported the abnormal appearance of the cuneus in people with ASD. Based on fMRI, Wichers et al. (2021) found that during sustained attention tasks, compared to controls, adults with ASD had decreased brain activation in the right cuneus. Guo detected that the decrease in ALFF was significant for ASD in adolescents in the right cuneus (Guo et al., 2017). In a study by Redcay and Courchesne (2008), they also found that infants with ASD (2–3-year-old children) had reduced activity in the right cuneus. Our results supplement the study of right cuneus at different stages of development for ASD. Combined with the results of previous studies, we hypothesized that decreased activity of the right cuneus may begin in infant ASD and persist throughout the patient’s all stages of development.

From the perspective of the overall analysis of the whole-brain function of prepubertal ASD patients, unlike the research results about ASD in the infant stage (Redcay and Courchesne, 2008), preschool stage (Lan et al., 2021), and adolescent period (Shukla et al., 2010), we did not find any regions that had increased brain activity. This is similar to brain development. Research has shown that infancy and preschool are two periods of peak brain development, followed by a decrease in brain growth, which is in preparation for the third peak at the beginning of puberty (National Research and Institute of Medicine Committee on Integrating the Science of Early Childhood, 2000). We hypothesized that the general decrease in brain activity in prepubertal ASD patients may be related to the reduced speed of brain development at this stage, and this may be a unique feature of prepubertal ASD patients. However, the specific mechanism needs to be further studied.

ALFF and ReHo are two important indicators demonstrating neural intensity and neural coherence. Previous work suggests that there is a functional correlation between ReHo and ALFF in the abnormal brain function region of some diseases (Yuan et al., 2013; Zhu et al., 2015; Xu et al., 2020). In this study, we found no correlation between ReHo and ALFF indicators in abnormal brain functional areas. This indicates that the assessment of prepubertal ASD patients needs to be conducted from ALFF and ReHo dimensions, respectively.

There are some limitations in our study. First, the sample size is limited, perhaps providing insufficient statistical power to detect more modest alterations, making the correlation between ReHo value in abnormal brain regions and ADOS subscore not solid. Studies with larger samples are required. Besides, in the future, as the sample size increases, we will adopt more advanced machine learning methods such as autoencoders (AE), long short-term memory (LSTM), recurrent neural network (RNN), deep belief network (DBN), and convolutional neural network (CNN) combined with the ALFF, REHO to study the prepubertal boys with ASD. Second, the brain functional alterations in this study were confined to the cerebrum. Other brain regions, such as the cerebellum which may also play an essential role in the pathophysiology of clinical functional impairment in ASD patients were not considered. Future research involving other brain functional regions is warranted. Third, we investigated ALFF/ReHo only in boys with ASD, and the interpretation of our results cannot be extended to female individuals with ASD.

## Conclusion

Based on RS-fMRI data, brain regions with functional abnormalities, including the left IPL, left LG, left STG, left MOG, and right cuneus may be crucial in the neuropathology of prepubertal boys with ASD. Furthermore, our findings indicated that ReHo abnormalities in the left LG and left STG were correlated with the ADOS score. These results will supplement the study of neural mechanisms in ASD at the different developmental stages and will be helpful to explore the abnormal sociality of prepubertal boys with ASD.

## Data Availability Statement

The raw data supporting the conclusions of this article will be made available by the authors, without undue reservation.

## Ethics Statement

The data was obtained from the public database. According to the policy of the institutional ethical review board, this kind of research is waived for ethical review.

## Author Contributions

XY: validation, formal analysis, investigation, and writing – original draft. GZ and XL: methodology. YS and YB: software. WW, YL, and HW: validation. ZL and XZ: data curation. MW: formal analysis, data curation, and writing – review and editing. All authors contributed to the article and approved the submitted version.

## Conflict of Interest

XZ was employed by Siemens Healthineers Ltd. The remaining authors declare that the research was conducted in the absence of any commercial or financial relationships that could be construed as a potential conflict of interest.

## Publisher’s Note

All claims expressed in this article are solely those of the authors and do not necessarily represent those of their affiliated organizations, or those of the publisher, the editors and the reviewers. Any product that may be evaluated in this article, or claim that may be made by its manufacturer, is not guaranteed or endorsed by the publisher.

## Footnotes

1
http://fcon_1000.projects.nitrc.org/indi/abide/
